# Expanding Ocular Care in the Emergency Department: A Comprehensive Review of the Utility of Point-of-Care Ultrasound for Ocular Emergencies

**DOI:** 10.7759/cureus.74548

**Published:** 2024-11-27

**Authors:** Rola R Alsulami, Jawza A Almutairi, Mohammad I Almatrafi, Basant S Othman

**Affiliations:** 1 College of Medicine and Surgery, Umm Al-Qura University, Makkah, SAU; 2 Department of Ophthalmology, King Saud Medical City, Riyadh, SAU; 3 Department of Ophthalmology, Al Noor Specialized Hospital, Makkah, SAU

**Keywords:** emergency department, ocular emergency, ocular ultrasound, point-of-care-ultrasound, retinal detachment (rd)

## Abstract

A time-sensitive, sight-threatening ocular condition presenting at an emergency department can be safely diagnosed promptly by ocular ultrasonography (OUS). OUS is a quick, safe, and portable option for assessing severe tissue damage to the periorbital area. OUS can help identify patients who need immediate ophthalmology consultation in a busy setting or when direct ophthalmic examination is unavailable. This review describes the use of OUS to diagnose ocular emergencies, including the indications, anatomy, and conditions (e.g., retinal detachment, vitreous hemorrhage, papilledema, and endophthalmitis) for OUS. Based on the current literature, OUS shows high sensitivity and specificity. Future research should focus on standardizing the use of OUS in clinical practice.

## Introduction and background

Point-of-care ultrasound (POCUS) has many advantages and is indicated in patients with trauma to the abdomen, chest, pelvis, and extremities [1]. Ocular injuries, ranging from conjunctivitis to vision-threatening causes, account for approximately 3% of all emergency department (ED) visits [2,3]. Direct examination and orbital computed tomography (CT) are utilized to evaluate such cases. However, when there is severe tissue damage to the periorbital area, direct ophthalmic examination might be difficult and cannot be performed at the bedside. In such situations, ocular ultrasonography (OUS) is quick, safe, and portable [4,5]. OUS can show the structures of the posterior chamber when cataract, hyphema, or periorbital edema makes its visualization impossible [6]. POCUS can diagnose many ocular conditions in emergency settings, including periorbital or intraocular foreign bodies (IOFBs), retinal or vitreous detachment, central retinal artery occlusion (CRAO), lens subluxation, papilledema [3,7], and retinoblastoma [8,9].

Research on the use of OUS for detecting retinal detachment (RD) has demonstrated a sensitivity ranging from 97% to 100% and specificity between 83% and 96% [10-12]. Furthermore, OUS is also effective in identifying vitreous hemorrhage (VH) and detachment, with reported sensitivity and specificity of 81.42% and 82.96%, respectively [13].

OUS can be used in EDs to facilitate the recognition of sight-threatening conditions. Emergency physicians who are trained to use OUS can detect many ophthalmic conditions accurately [6]. This can help identify patients who need immediate ophthalmology consultation in a busy ED. Based on the previous literature, the current work aimed to review the use of POCUS for ocular emergencies.

## Review

Methods

A comprehensive search of several databases, including PubMed, Google Scholar, and Web of Science, was conducted between September and October 2023. The search was limited to English-language, human-based trials and articles published within the past 15 years. Several mesh terms and their combinations were used with the Boolean operators to search for the related article. The mesh terms used included “point of care ultrasound”, “POCUS”, “ocular emergencies”, “emergency department”, “RD”, “CRAO”, “orbital vasculature”, “optic nerve”, “papilledema”, “optic disc swelling”, “intra-orbital foreign body”, “intra-ocular foreign body”, “vitreous hemorrhage”, “bulbar hemorrhage”, “endophthalmitis”, and “ruptured globe”. All retrospective and prospective observational studies, systematic reviews and meta-analyses, case reports, case studies, and relevant guidelines were included in this review. Non-English articles, letters, and editorials were excluded.

Discussion

Overview of Ocular POCUS

Ocular POCUS is frequently used in ED to evaluate various ocular conditions such as trauma, RD, CRAO, papilledema, hyphema, and the presence of IOFBs, and to assess intracranial pressure (ICP) [14-16]. It serves as a valuable diagnostic tool, enabling emergency physicians to quickly and accurately diagnose ocular disorders [14,17,18]. A recent meta-analysis emphasized its strong diagnostic accuracy, particularly for posterior chamber abnormalities that are often encountered in the ED [15]. However, POCUS is contraindicated when there is a suspicion of globe rupture due to the potential risk it poses [19,20].

**Figure 1:**
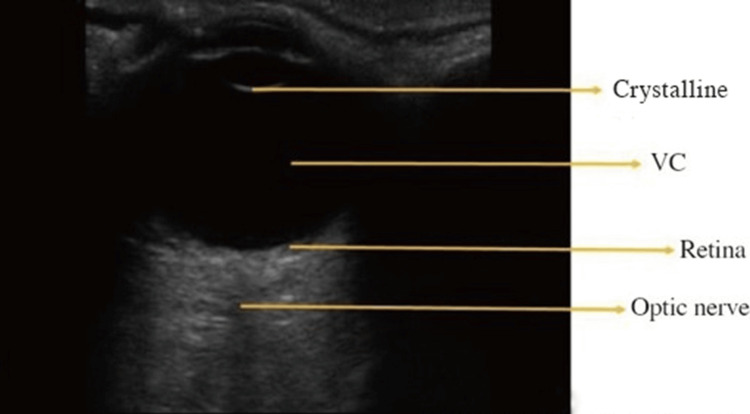
Normal eye VC: vitreous chamber Credits: Permission was obtained from the original authors of the images Reference: [7]

On POCUS, different ocular structures have distinct appearances. The retina, choroid, and sclera present as a single curved echogenic line [21], while both lens capsules are echogenic, and the interior of the lens appears anechoic [15,22]. The average distance from the cornea to the retina is about 24 mm [10]. Extraorbital structures also differ in their ultrasound appearance; retrobulbar fat is hyperechoic, whereas the optic nerve and muscles are hypoechoic [20,23] (Figure 1).

**Figure 2:**
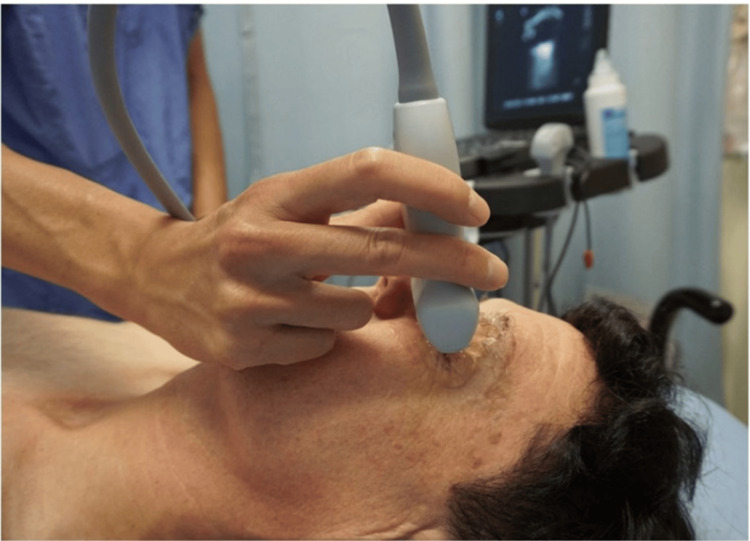
Application method Credits: Permission was obtained from the original authors of the images Reference: [29]

The technique for obtaining ultrasound images of the eye was first described in 1987 [24]. It involves applying a transparent adhesive dressing to the patient's eye, followed by a layer of gel to the closed eyelid, which serves as a transducer interface [25]. A comprehensive examination of the orbit requires scanning the entire structure. A linear probe is commonly used to capture sagittal and transverse views, and positioning the examiner's hand against the patient's nose or cheek can help minimize pressure and enhance stability [26-28]. To further reduce the sonographic energy used, specific ocular ultrasound settings are applied to the machine (Figure 2) [29,30].

Other Ocular Emergencies

Ocular trauma (OT): OT constitutes the majority of ED eye-related complaints, accounting for approximately 78% of all cases [20,31]. OT ranges from contusions to lacerations, fractures of the orbital wall, superficial abrasions, ruptured globes, and IOFBs [31]. Each condition can be associated with posterior injuries, which can be detected by OUS. When a ruptured globe is suspected, further manipulation of the globe should be avoided. However, in certain situations, the examination is limited due to periorbital swelling or tissue damage. OUS can be used to examine the posterior portion of the eye. The findings that suggest a ruptured globe are orbital compression, loss of volume, or a heterogeneous blood and vitreous mixture in the posterior segment [32]. IOFBs can be detected by OUS and appear as reverberations and comet tail artifacts. However, when the foreign body is seen, the examination should stop immediately, as it is associated with a ruptured globe [33]. Bleeding in a closed space tends to increase pressure; similarly, patients with retrobulbar hemorrhage present with pain due to increased intra-compartmental pressure. In OUS, retrobulbar hemorrhage appears as anechoic fluid collecting behind the eye resembling a guitar pick, which is seen when the posterior globe undergoes conical deformation [21,34].

RD: RD is a serious ocular emergency that requires prompt identification to avoid permanent vision impairment [20]. RD occurs when the neurosensory retina separates from the underlying pigmented epithelium [7]. Patients typically present with symptoms such as new floaters, flashes of light, or sudden loss of vision in one eye, often described as a curtain descending over their field of view.

POCUS has proven to be a reliable, fast, and non-invasive method for diagnosing RD. Individuals diagnosed with RD should be promptly referred to an ophthalmologist for a more thorough evaluation and treatment to prevent lasting vision loss [20]. A 2020 meta-analysis found that ED physicians can confidently rule out RD if POCUS results are negative [21].

**Figure 3:**
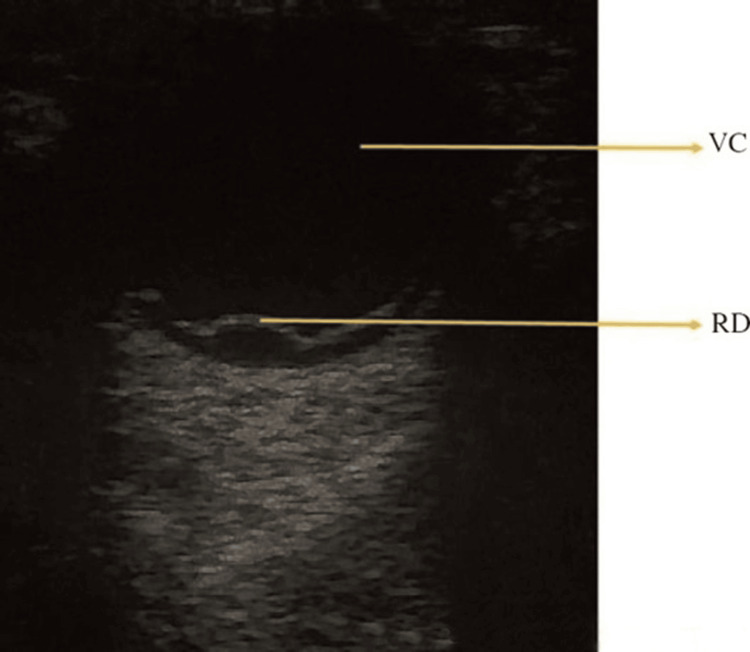
Retinal detachment VC: vitreous chamber; RD: retinal detachment Credits: Permission was obtained from the original authors of the images Reference: [7]

In a prospective study of 225 patients with symptoms suggestive of RD, POCUS demonstrated a sensitivity of 96.9% and a specificity of 88.1% for detecting RD [13]. B-mode ultrasonography was used to assess the vitreous body and posterior chamber, with RD identified by a distinct echogenic membrane tethered to the optic disc and separating from the choroid. RD was distinguished from vitreous detachment based on the membrane’s appearance and its tethering to the optic nerve (Figure 3) [13].

VH: VH refers to the presence of blood within the vitreous cavity, which is bordered posteriorly and laterally by the retina's internal limiting membrane, laterally by the non-pigmented epithelium of the ciliary body, and anteriorly by the zonular fibers and posterior capsule of the lens [13]. VH can result from disruption of retinal vessels, hemorrhage, or abnormal blood vessel growth (angiogenesis) [35,36]. Risk factors for VH include systemic conditions like diabetes, hypertension, stroke, and blood disorders such as hemophilia and polycythemia, as well as ocular conditions like trauma, uveitis, posterior vitreous detachment, and RD [37]. Clinically, VH typically presents as a sudden, painless reduction in vision in one eye, and patients may also experience flashes of light (photopsia) [36,37].

**Figure 4:**
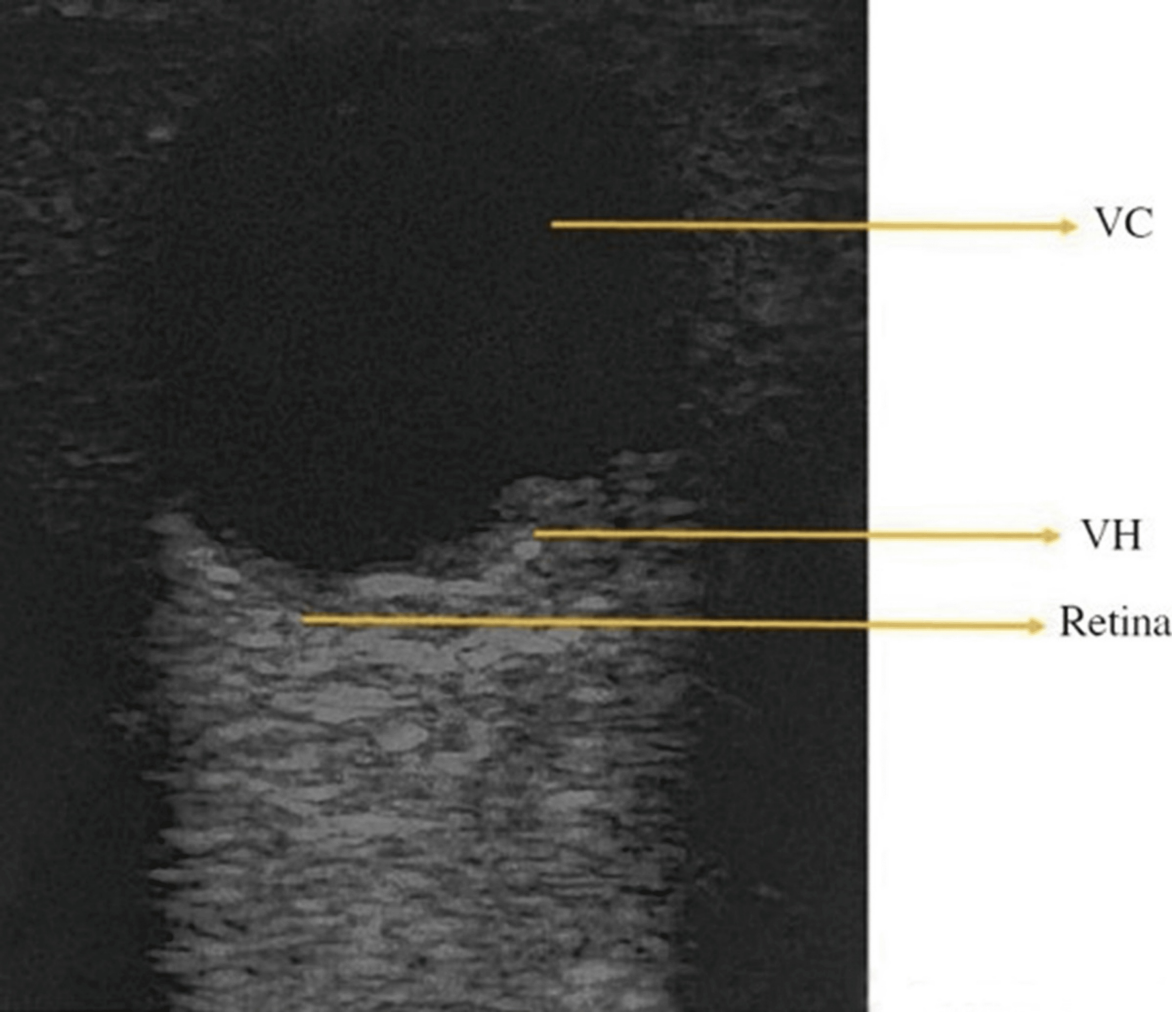
Vitreous hemorrhage VC: vitreous chamber; VH: vitreous hemorrhage Credits: Permission was obtained from the original authors of the images Reference: [7]

Though VH can occur alongside retinal tears, it is generally not considered an immediate emergency. Treatment can often be delayed to allow monitoring of blood clearance and management of any underlying conditions [38]. In a systematic review and meta-analysis, Propst et al. [21] found that POCUS had moderate diagnostic accuracy for detecting VH, with a sensitivity of 90% (95% CI, 65-98%) and specificity of 92% (95% CI, 75-98%). Additionally, a prospective study conducted in four EDs involving 225 patients found that ocular POCUS had a sensitivity of 81.9% and specificity of 82.3% for diagnosing VH [13]. VH typically appears as a hazy, hyperechoic area in the posterior segment during the POCUS examination (Figure 4) [39].

CRAO: CRAO is a leading cause of irreversible vision loss. It is an acute stroke of the eye and is associated with a high risk of cerebrovascular assault and ischemic heart disease [40]. Patients with CRAO need combined management between neurology and ophthalmology [41]. Unilateral painless loss of vision with temporal sparing is the usual manifestation of CRAO [42].

**Figure 5:**
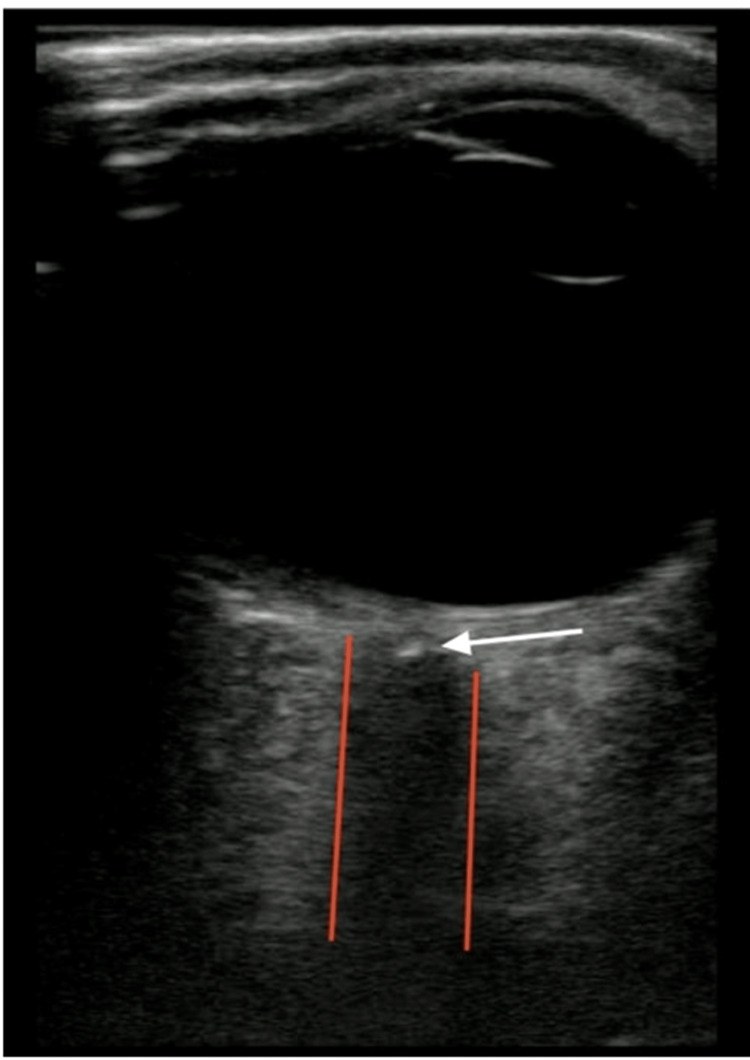
Central retinal artery occlusion (CRAO) and spot sign Credits: Permission was obtained from the original authors of the images Reference: [43]

The POCUS is a fast and effective tool for assessing both arterial and venous flow in cases of retinal vessel compromise, using color Doppler imaging to visualize retinal artery thrombosis. Emergency physicians can use POCUS to rapidly diagnose CRAO, which may reduce the time to treatment and improve the chances of a better visual outcome [43]. The technique for detecting CRAO with POCUS is similar to that for RD, involving the placement of the transducer on a closed eyelid. CRAO presents as a hyperechoic signal in the optic nerve, known as the retrobulbar spot sign (RBSS) (Figure 5) [20].

ICPA retrospective case series of nine patients with an average vision loss duration of 21 hours found that 88% were diagnosed with CRAO, and 75% had POCUS-confirmed RBSS, indicative of CRAO [43]. The authors concluded that ocular POCUS enables the rapid diagnosis of CRAO, thereby increasing the chances of better visual outcomes. The detection of RBSS with ocular POCUS demonstrated 100% specificity for CRAO [44].

**Figure 6:**
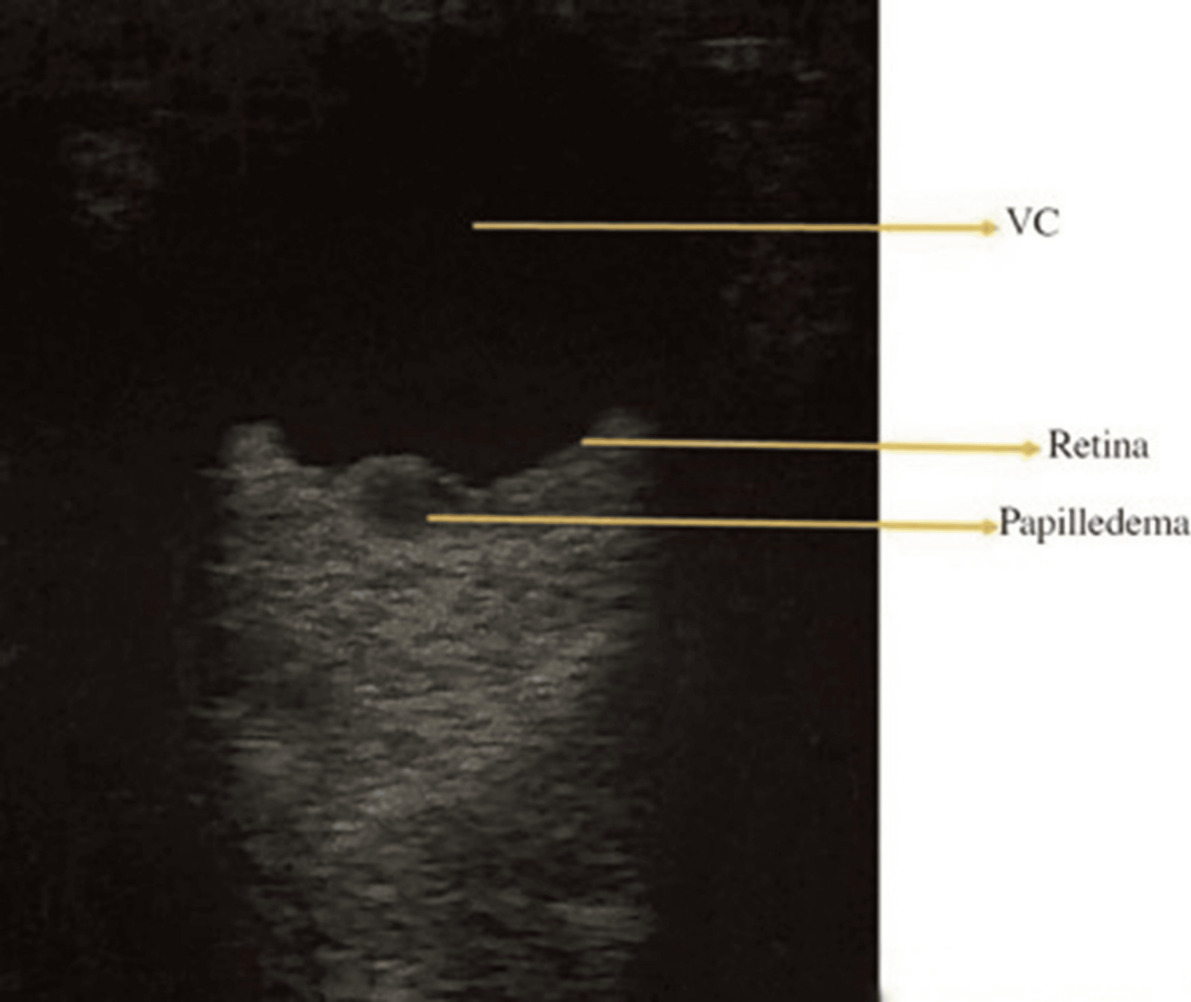
Papilledema VC: vitreous chamber Credits: Permission was obtained from the original authors of the images Reference: [7]

Papilledema: Papilledema refers to optic disc swelling caused by elevated ICP (EICP) [7]. While typically bilateral, it can sometimes present asymmetrically or, in rare cases, unilaterally [45]. Increased ICP impacts the subarachnoid space surrounding the optic nerve, disrupting its metabolic function and leading to progressive vision loss [46]. Clinical findings may include dilated retinal veins, disc hemorrhages, and hyperemia of the optic disc [47]. On ultrasonographic examination, papilledema is characterized by an elevated optic disc and an increase in optic nerve thickness (Figure 6) [41,48].

**Figure 7:**
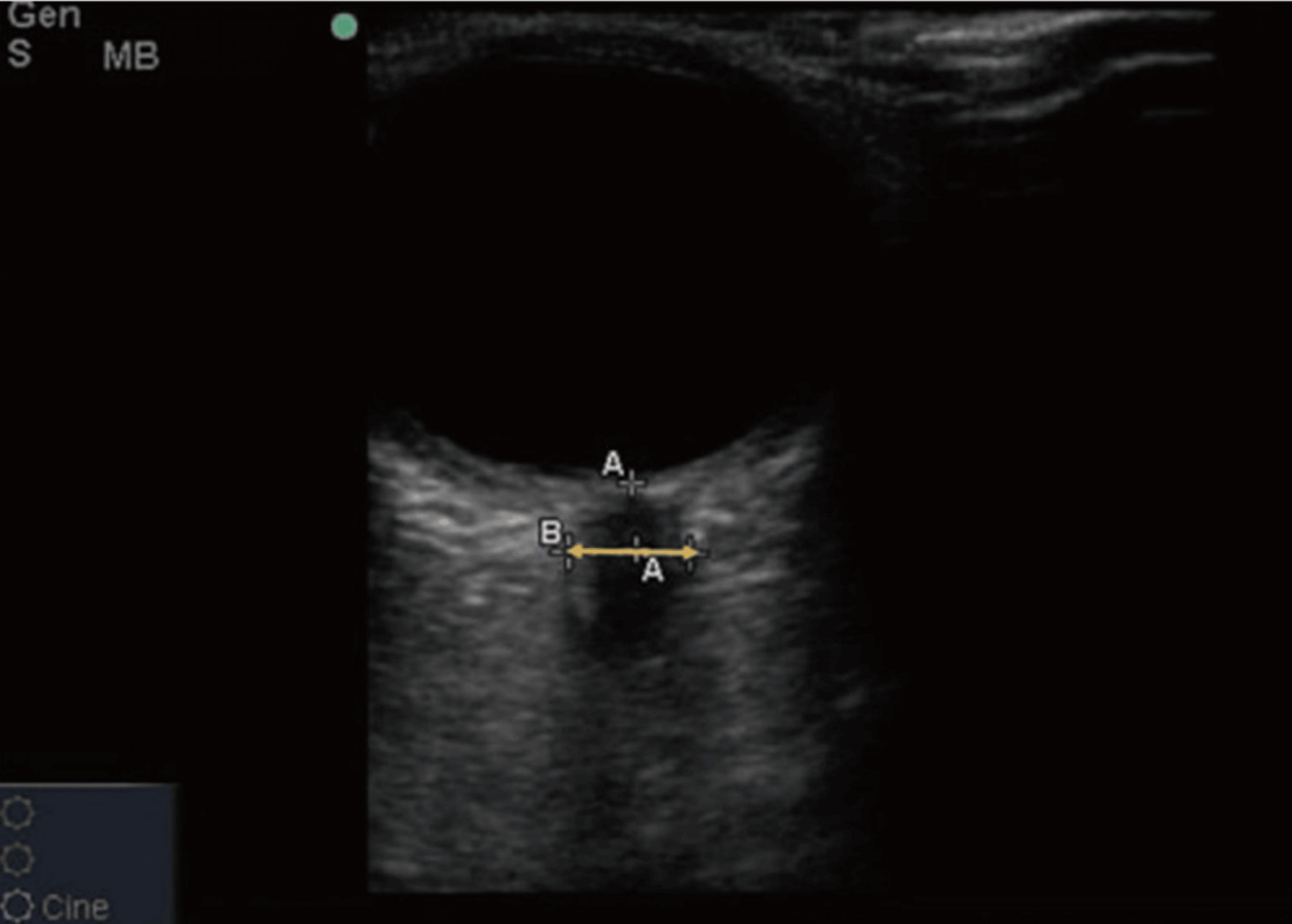
Increased thickness of the optic nerve A: 0.30 cm; B: 0.55 cm Credits: Permission was obtained from the original authors of the images Reference: [7]

There is growing evidence supporting the use of ultrasound to measure the optic nerve sheath diameter (ONSD) as a sensitive and effective method for detecting EICP. In their pioneering study, Hansen and Helmke [49] demonstrated a correlation between increased cerebrospinal fluid pressure and ONSD. Blaivas et al. [3] suggested a 5 mm ONSD cutoff as a sensitive marker for detecting EICP based on CT findings. Various studies have reported that ONSD measurements, with cutoffs ranging from 4.8 to 5.9 mm, align well with invasive ICP monitoring [48-53]. An ONSD measurement greater than 5 mm, typically taken 3 mm behind the orbit, has been shown to be a sensitive (88%) and specific (93%) indicator of EICP [54-57]. Moretti et al. [50] reported a 5.2 mm cutoff with 93% sensitivity and 74% specificity, while Rajajee et al. [58] found a 4.8 mm cutoff to have 96% sensitivity and 94% specificity. Additionally, measuring an optic disc height of 0.6 mm offers 91% sensitivity and 75% specificity for diagnosing papilledema [47,48]. The height of the optic disc, from its peak to where it intersects with the posterior globe, can be assessed using ultrasound. This method of measuring ONSD via ultrasonography provides a simple and reliable means of detecting increased ICP (Figure 7) [53,58].

IOFBs: IOFBs have definite diagnostic and management challenges, as they might not be obvious in the initial assessment and can result in sight-threatening complications. Therefore, IOFBs require a high index of suspension to prevent the occurrence of permanent vision loss due to short-term complications such as direct injuries, endophthalmitis, and RD and late-onset complications such as siderosis bulbi.

**Figure 8:**
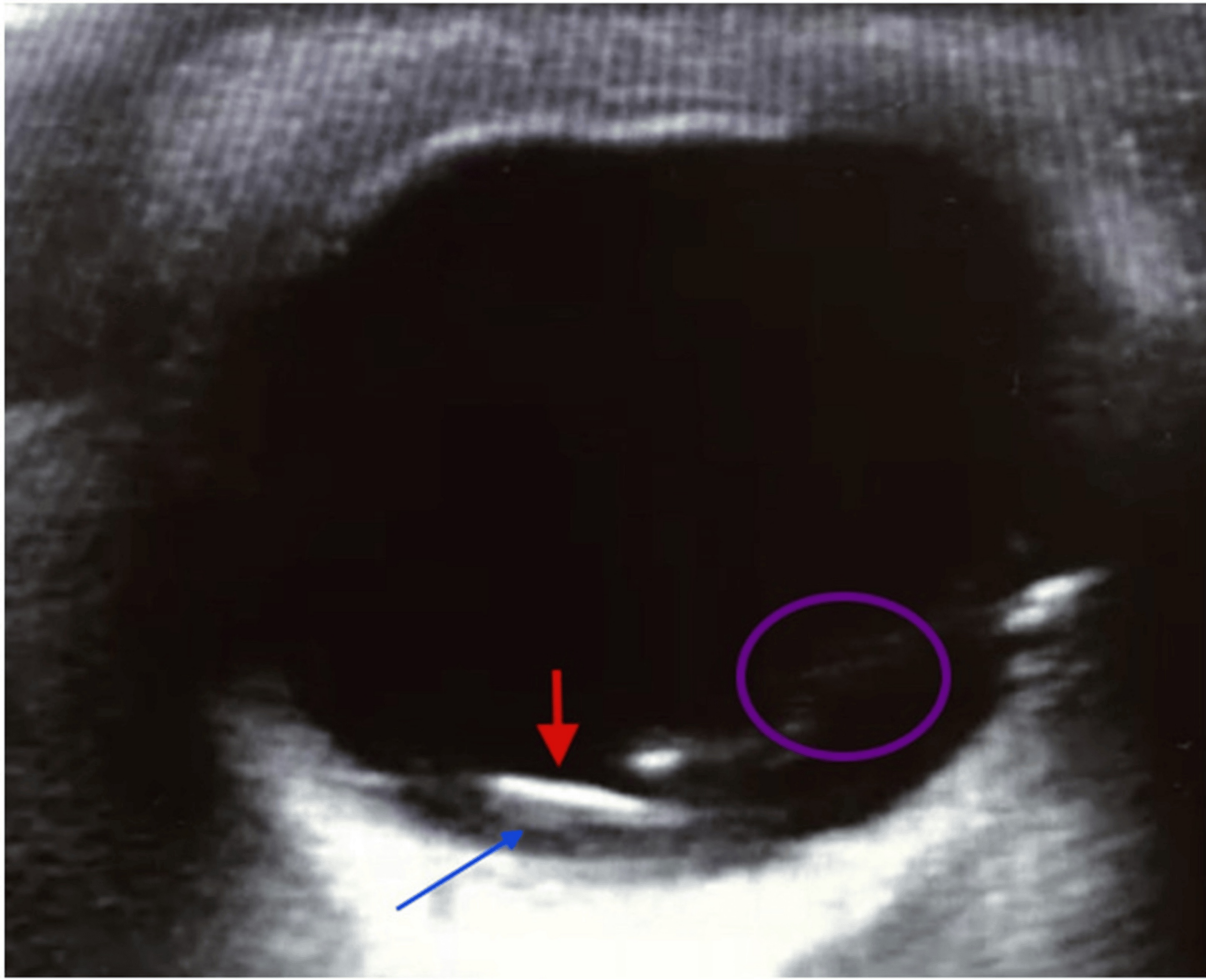
Intraocular foreign bodies (IOFBs) Radiolucent hyperechoic structure (red arrow) with reverberation within the posterior chamber (blue arrow). Credits: Permission was obtained from the original authors of the images Reference: [59]

Identification of IOFBs is traditionally done by orbital CT. However, due to CT’s limited availability and the risk of exposure to high doses of radiation, POCUS is a quick, reliable, and important alternative to orbital CT. In a case report of a young male presenting with OT, POCUS showed a mobile, non-radiopaque hyperechoic structure, most likely a metallic FB with associated linear areas likely representing VH [59]. Orbital CT was performed and showed findings consistent with POCUS (Figure 8).

IOFBs can be easily missed, as the patient might be completely asymptomatic with a small entry wound as the only presenting finding. POCUS is of high importance in detecting these IOFBs, and their presence necessitates early surgical intervention in more than 90% of cases [59].

Other

The POCUS is an effective tool for detecting ocular infections, such as endophthalmitis, a bacterial or fungal infection affecting the vitreous and/or aqueous humor [60]. This is one of the most severe eye infections, potentially leading to irreversible blindness in the affected eye within hours or days after symptoms appear. Tucker et al. [61] described a case where POCUS was used to identify suspected exogenous endophthalmitis in a patient who had recently undergone cataract surgery. Bedside ultrasound showed numerous mobile, hyperechoic densities throughout the globe. Another case report echoed these findings, revealing significant, well-defined, mobile hyperechoic debris in the posterior chamber [62]. Additionally, hyperechoic material was detected in the lens, heightening concerns about endophthalmitis and leading to a swift diagnosis and timely management of this sight-threatening condition [62].

Limitations of POCUS

A limitation of POCUS is operator dependency, as non-well-skilled emergency practitioners might not be able to diagnose sight-threatening conditions, which could result in a misdiagnosis and affect the visual outcome of the patient [63]. The safety concern is another limitation, especially in traumatic ocular injuries, where minimal and gentle digital handling of the globe is a must to prevent further damage to intraorbital structures [1]. Furthermore, POCUS cannot comprehensively evaluate different ocular emergencies, which must be combined with more specialized ophthalmological assessment tools [12,20].

Future directions of POCUS

Training programs targeting EPs would help to improve their skills and confidence in diagnosing common ocular emergencies using POCUS [20]. Enhancing the availability of portable and cost-effective POCUS machines at different EDs, especially in resource-limited areas, would improve EPs’ access and allow them to master the necessary skills for the diagnosis of different ocular emergencies [20]. Artificial intelligence can assist in image interpretation and diagnosis, potentially improving the accuracy of POCUS assessment. Integrating POCUS into telemedicine can enable remote consultations with an ophthalmologist, allowing for rapid assessment and guidance in ocular emergencies, particularly in areas where an on-call ophthalmologist is not available [64].

## Conclusions

POCUS is a useful tool for diagnosing ophthalmic emergencies and can prevent serious complications and improve the overall outcome of these conditions. OUS is used to diagnose posterior globe injuries and globe compression. In addition, B-mode ultrasonography is used in cases of RD, as the detachment appears as a bright echogenic membrane. In the vitreous cavity, POCUS reveals materials in the posterior chambers that shift with eye movement. CRVO appears as a hyperechoic signal in the middle of the optic nerve. Papilledema is diagnosed by measuring the optic sheath diameter. Endophthalmitis presents multiple hyperechoic densities throughout the globe.
